# Extreme risk protection orders and firearm violence: a synthetic control analysis

**DOI:** 10.1186/s40621-026-00663-8

**Published:** 2026-02-16

**Authors:** Hannah Rochford, Vivek Ashok

**Affiliations:** 1 School of Public Health Administration Building, Department of Health Policy & Management, Texas A&M University, 212 Adriance Lab Rd, Office 116, College Station, TX 77843 USA; 2 Division of General and Community Pediatrics, Cincinnati Children’s Hospital Medical Center, Cincinnati, OH USA; 3 Department of Pediatrics, University of Cincinnati, Cincinnati, OH USA; 4 The Children’s Hospital of Philadelphia, PolicyLab, Philadelphia, PA USA

**Keywords:** Firearm injury, Firearm violence, Extreme risk protection orders, ERPOs, Synthetic control

## Abstract

**Background:**

Extreme Risk Protection Orders (ERPOs) are an evidence-based provision to prevent firearm violence present in 21 states, and Washington D.C. as of 2024. Examining the potential of varied ERPO versions to prevent fatal and nonfatal forms of firearm violence is crucial for shaping effective policy creation and enactment.

**Methods:**

We use a synthetic control approach to estimate how varied ERPO versions impact firearm violence incidents resulting in injury and/or death per the Gun Violence Archive between 2014 and 2021. Our ‘treated’ state cohort (California, Delaware, Florida, Illinois, Massachusetts, Maryland, Oregon, Rhode Island, Vermont, and Washington) had ERPO effective dates after 2015 and before 2019, and experienced a statistically significant increase in petitions relative to the petition volume (zero) before ERPO implementation.

**Results:**

Significant reductions in state-month firearm violence rates were associated with ERPO policies in Rhode Island and Massachusetts (due largely to reductions in nonfatal firearm assault). Florida’s, Oregon’s, Vermont’s, and Washington’s ERPO policies were not associated with changes in firearm violence incidents. Poor pre-period fit made California, Delaware, Illinois, and Maryland results uninterpretable.

**Conclusions:**

We conclude not all ERPO policies are created or implemented equally. Under certain enactment and implementation conditions, ERPO policies may protect against nonfatal firearm assault incidents in particular. Increasing effect sizes over time may suggest state capacity for implementing ERPOs effectively is improving, however, effect size magnitudes also indicate factors other than ERPO policies may be contributing to observed declines.

**Supplementary Information:**

The online version contains supplementary material available at 10.1186/s40621-026-00663-8.

## Introduction

Firearms caused 46,728 deaths in the US in 2023 [1]. Nonfatal firearm injuries occur at more than twice the rate of firearm fatalities [2], and often launch an expensive sequela of long-term mental and physical health morbidities for survivors, families, and communities [3]. In 2020, the estimated combined lifetime costs for firearm-related deaths and annual costs of nonfatal firearm injuries tallied $493.2 billion [4]. 

All forms of firearm injury are preventable. These incidents are heavily shaped by structural factors including policies regulating firearm access, ownership, and availability. States with a higher number of firearm regulations and more stringent policies are associated with population-level reductions in firearm injury [5–7]. 

Extreme Risk Protection Orders (ERPOs) are one policy approach intended to prevent firearm injury. Enacted in 21 states and Washington D.C. as of 2024, ERPOs, or “Red Flag” laws, temporarily restrict firearm access for individuals at risk for harming themselves or others. The first four states to adopt ERPOs were Connecticut (1999), Indiana (2005), California and Washington (2016). All other states with ERPOs (Colorado, Delaware, Florida, Hawaii, Illinois, Maryland, Massachusetts, Michigan, Minnesota, Nevada, New Jersey, New Mexico, New York, Oregon, Rhode Island, Vermont, Virginia, and Washington D.C.) enacted these between 2018 and 2024.

States without ERPOs have other mechanisms for temporary removal of firearms if certain characteristics are met including a violent misdemeanor conviction, mental illness, or domestic violence. For instance, states vary in their laws restricting firearm access for individuals with serious mental illness, including provisions for firearm dispossession during mental health holds and/or involuntary civil commitments [8]. Involuntary civil commitments involve both the removal of firearms and restrictions on future firearm purchases, but they also impose broader limitations on civil liberties, such as involuntary psychiatric evaluation and potential detention [9]. Additionally, all 50 states and the D.C. have Domestic Violence Protective (Restraining) Orders which require perpetrators to surrender any firearms and prevents purchasing of new firearms [10]. 

ERPOs can complement and strengthen existing laws by providing a targeted mechanism focused exclusively on firearm access, rather than broader restrictions on civil liberties. They address specific instances of risk for potential violence rather than categorical individual characteristics, enable timely crisis intervention through ex parte due process, and permit petitions from a range of individuals beyond medical professionals, such as law enforcement and family members [9]. 

While research on ERPO effectiveness is ongoing, prior ERPO policy evaluations have focused largely on their effectiveness in reducing rates of firearm suicide, with descriptive studies highlighting their potential to reduce mass shootings [11–14]. For example, Swanson et al. estimated that in Connecticut, one death by firearm suicide would be prevented for every 10–20 ERPOs filed [13]. Similarly, Miller et al. estimated one suicide death was prevented for every 22 ERPOs filed [14]. ERPO’s impact on fatal firearm injuries, specifically firearm suicide, are encouraging though generalizability of results are limited by methodological constraints. For instance, studies evaluating the impact of ERPOs on firearm suicide are limited to single or two state analyses. Delafave assessed the impact of ERPOs on total and firearm homicides though log-linear two-way fixed-effects modeling; however, generalizability of results was limited due to gaps in data – specifically, exclusion of state-year observations with suppressed mortality data [15]. Additionally, varied study design, variations in statewide implementation, and recency of law passage in most states have limited effectiveness of research in this area.

Furthermore, studies assessing the impact of ERPOs on nonfatal firearm injuries remain limited. Pear et al. evaluated the effect of ERPO implementation in San Diego on annual county-level firearm assault rates, using hospitalization and mortality data from 2005 to 2016. The investigators applied a synthetic control approach, comparing San Diego to a counterfactual created from other California counties [16]. The study did not find a statistically significant effect, although this may reflect the inability to detect an effect at the population level rather than the absence of one.

Research examining the impact of firearm restrictive laws on nonfatal firearm injury is also limited. Two published studies have examined this issue: one assessed the impact of California’s policy regarding open carry handgun policy on nonfatal firearm-related injuries while another evaluated the effects of 12 firearm restrictive laws (none were ERPOs) on inpatient hospitalizations for firearm injury [17]. To address this gap, we used state-specific synthetic control modeling to estimate the impact of state ERPO laws on firearm violence incidents resulting in fatal or nonfatal injury.

## Methods

### Design and sample

In this state-month level synthetic control study, we estimate the impact of ERPO provisions on firearm incidents resulting in at least one death and/or one injury between 2014 and 2021. Explanatory data of interest were ERPO provisions enacted across states between January 1, 2015 - January 1, 2019 to allow for adequate pre- and post-period durations. These were derived from the National ERPO Resource Center [18], a project by the Center for Gun Violence Solutions within Johns Hopkins Bloomberg School of Public Health.

### Participants

States included in our treatment group met three criteria. First, they enacted an ERPO law prior to 2019 to allow for a sufficient post-policy period (excluded Hawaii, New Jersey, New Mexico, Nevada, New York, Virginia). Second, states enacted their ERPO after 2014 to allow for a sufficient pre-policy period (excluded Connecticut, and Indiana). Third, states must have experienced a statistically significant increase in ERPO petitions, determined by a t-test following enactment (excluded Colorado; See Supplement A). Petition volumes were accessed from Everytown for Gun Safety [19]. 

**Fig. 1:**
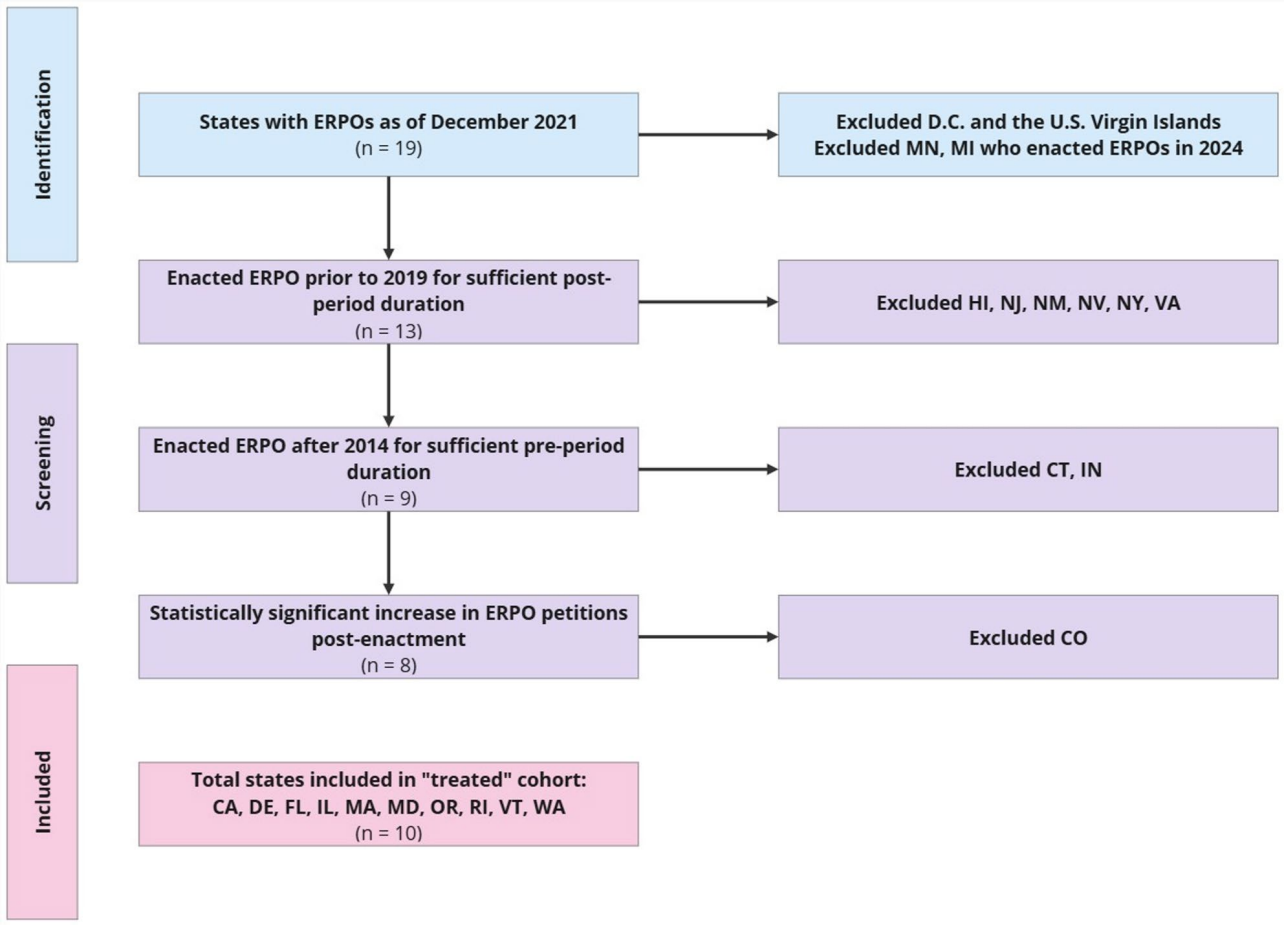
Treated state inclusion-exclusion summary

The final ‘treated’ cohort included California, Delaware, Florida, Illinois, Massachusetts, Maryland, Oregon, Rhode Island, Vermont, and Washington (Fig. 1). The control group, also known as the donor pool, included states that had not enacted ERPO laws during the study period and had no missing years of covariate data (Alabama, Alaska, Arizona, Arkansas, Georgia, Iowa, Indiana, Kansas, Kentucky, Louisiana, Michigan, Minnesota, Mississippi, Missouri, Montana, Nebraska, North Carolina, Ohio, Oklahoma, Pennsylvania, South Carolina, Tennessee, Utah, West Virginia, Wisconsin).

### Outcome data

The outcome of interest was state-month rates of firearm violence events resulting in at least one death or injury between 2014 and 2021. We obtained firearm violence incidents from 2014 to 2021 from the Gun Violence Archives (GVA). GVA defines a firearm violence incident as those involving firearm-related injuries or deaths, including assaults/homicide, self-directed violence/suicide, mass shootings, and unintentional injuries. GVA defines a firearm-related injury as a physical injury resulting directly from a projectile (i.e., bullet) discharged from a powder-actuated firearm. Injuries resulting from other mechanisms, such as blunt-force trauma involving a firearm or secondary fragmentation injuries caused by surfaces or objects struck by a bullet, are not included. The GVA also excludes injuries associated with non-powder or low-velocity firearms (e.g., BB, pellet, or air guns).The database, compiled daily through automated queries and manual searches of media reports, law enforcement sources, government databases, and data aggregates, has been validated by prior research for epidemiologic research and found to have strong sensitivity (81.1%) and near perfect positive predictive value (99.0%) [20]. Each GVA incident includes the date, location (street address), number of victims and suspects injured or killed, and source links. Observations without reported injuries or deaths (e.g., warning shots, robberies involving guns, police discovering guns in searches for illicit substances), were excluded from our analysis [20]. While more recent GVA data is available, we restricted our sample to span 2014 to 2021 to avoid biases introduced by a 2022 GVA methodological shift in capturing self-directed violence.

### Covariate data

In alignment with recent recommendations for addressing confounding when estimating the impact of firearm policy [21], we included the following covariates: state-year indicators for Republican control of legislative houses and the executive branch (scored 0–3); incarceration rate; the proportion of wealth held by the wealthiest decile; average household income; unemployment, uninsured, and poverty rates; proxied firearm ownership (measured as the proportion of suicides committed with a firearm), which has been correlated with state-specific measures of firearm ownership measures; [22, 23] and, sociodemographic composition (including the proportion of residents under 18, between 18 and 34, and over 65, who identify as white, who hold a Bachelor’s degree or higher, who are veterans, and who were born outside of the US).

We controlled for the state-year prevalence of mental and behavioral health challenges using Behavioral Risk Factor Surveillance System (BRFSS) data (the proportion of the state in the 80th percentile of self-reported alcohol consumption, and the proportion of the state with 14 or more “not good” mental health days in the last 30 days), and the presence of other policies impacting high-risk firearm possession (firearm possession prohibitions for individuals involuntarily committed to an inpatient facility, involuntarily committed to an outpatient facility, with alcoholism or with certain alcohol related problems, with drug misdemeanor convictions, with felony convictions, with a violent misdemeanor conviction punishable by less than a year in prison, and by more than one year in prison) per Dr. Michael Siegel’s firearm policy database [24]. Lastly, given that the period of interest included the height of the COVID-19 pandemic, we included state-month indicators for a state’s COVID-19 death rate [25] and for whether or not a lockdown was active [26]. See Supplement B for additional detail regarding explanatory, outcome, and covariate data.

### Statistical analysis

All analyses were performed in STATA 18.5. Abadie-Diamond-Hainmueller’s synthetic control approach was used to estimate the impact of ERPO enactment and fatal and nonfatal firearm-related incidents [27]. A detailed discussion of this method, all three inference tests used, and sample statistical code are available elsewhere [28]. Because of considerable state-to-state variation in ERPO implementation and uptake, and nuanced differences in ERPO policy specifications (e.g., burden of proof standards, number and types of eligible petitioners, duration and implementation support (Table 1), we generate state-specific impact estimates to avoid inappropriately equating varied ERPO policies.

**Table 1:** Summary of key ERPO provisions and effective dates for treated States

Effective date	California	Delaware^4^	Florida	Illinois	Massachusetts	Maryland	Oregon	Rhode Island	Vermont	Washington
	1 Jan 2016	27 Dec 2018	9 Mar 2018	1 Jan 2019	17 Aug 2018	1 Oct 2018	1 Jan 2018	1 Jun 2018	11 Apr 2018	8 Dec 2016
**Eligible petitioners**										
Number	4	2	1	2	2	3	2	1	2	2
Types	o Law Enforcement o Family or household member o Employer, or coworker, o Secondary and Post-secondary Teachers, or Post-Secondary Employees	o Law enforcement o Family member (can only petition for a non-emergency order)	o Law enforcement	o Law enforcement o Family or household member	o Law enforcement o Family or household member	o Law enforcement o Family or household member o Medical professional	o Law enforcement o Family or household member	o Law enforcement	o State’s Attorney o Office of the Attorney General	o Law enforcement o Family or household member
**Burden of proof** ^**1**^										
for Temporary^2^ Orders	Reasonable cause	Preponderance of evidence	Reasonable cause	Probable cause	Reasonable cause	Reasonable cause	Probable cause	Probable cause	Preponderance of evidence	Reasonable cause
for Final Orders	Clear & Convincing	Clear & Convincing	Clear & Convincing	Clear & Convincing	Preponderance of evidence	Clear & Convincing	Preponderance of evidence	Clear & Convincing	Clear & Convincing	Preponderance of evidence
**Duration**										
for Temporary^2^ Orders	Up to 21 days post issuance	Up to 10 days post issuance	Up to 14 days post issuance	Up to 14 days post issuance	Up to 10 days post issuance	Up to 7 days post service	Up to 30 days post service	Up to 14 days post issuance	14 days post issuance	Up to 14 days post issuance
for Final Orders	Up to 5 years	1 year	Up to 1 year	Up to 1 year	Up to 1 year	Up to 1 year	1 year	1 year	Up to 6 months	1 year
**Implementation considerations**										
Percent of counties reporting at least one ERPO filed since implementation^3^	90%	100%	94%	52%	-	100%	83%	100%	93%	87%
Second Amendment Sanctuary Counties^5^	7%	0%	66%	67%	0%	42%	69%	0%	0%	62%

^1^ Burden of proof range, in descending order from most challenging to reach to most feasible to reach: [1] beyond a reasonable doubt [2], clear and convincing [3], preponderance of evidence [4], probable cause [5], reasonable cause

^2^ Temporary orders encompass ‘temporary’, ‘emergency’, ‘ex parte’, and ‘interim’ orders

^3^ Everytown for Gun Safety. *Guide to Using Extreme Risk Orders to Save Lives.* Everytown Research & Policy. Published [year]. Accessed March 3, 2025. https://everytownresearch.org/guide-to-using-extreme-risk-orders-to-save-lives/

^4^ Since 2018, has had multiple revisions of the law to expand the list of petitioners, criteria for petitioning, and lethal means restriction (Erpo.org)

^5^ Per Knoepke CE, Barnard LM, Batta N, et al. Petitions for Extreme Risk Protection Orders and Second Amendment Sanctuary Status in Colorado. *JAMA Netw Open*. 2024;7 [4]:e244381. doi:10.1001/jamanetworkopen.2024.4381, ERPOs were still issued within Second Amendment counties in cases involving threats of self-harm, interpersonal violence, or mass violence. However, Second Amendment sanctuary jurisdictions were less likely to be initiated by law enforcement and less likely to result in an emergency order being granted. Therefore, while ERPOs are still filed in Second Amendment counties, some individuals eligible for ERPOs in these jurisdictions may not receive them, which could dilute policy effectiveness

Using the *synth* package (STATA 18.5), we created a “synthetic” treated state, or synthetic control, to use as our counterfactual estimate. Aset of non-negative weights that sum to one were assigned to the donor units (states without ERPO policies) such that the difference between the treated unit’s and the synthetic control’s outcome variable predictors are minimized within the pre-intervention period. This same weighted average is extended to the outcome variable predictors in the post-period. Our policy impact estimate is derived from the difference between the estimated outcomes of our synthetic control and observed outcomes of our treated state in each year of the post-intervention period.

To determine if our estimates were statistically significant, we performed inference tests for each treated state analysis. First, we graph the gap between observed and synthetic outcomes in the pre- and post-periods. A near-zero gap in the pre-policy period reflects a strong model fit. In the post-policy period, a non-zero gap indicates a potential policy effect. Next, we compared the effect size of the actual treated unit to those generated for each donor unit’s to assess whether the observed change in the treated state exceeded what would be expected by chance among untreated donor units (placebos). We compiled graphs depicting the gaps between observed and synthetic outcomes for each placebo against our actual treated unit. To generate indicators of statistical significance, we calculated the root mean square prediction error (RMSPE) for each unit in the pre- and post-policy periods and computed the ratio of post- to pre-policy RMSPE. A ratio greater than one for the treated unit suggests a policy effect, while placebo units, having no intervention, should exhibit ratios near one. We ranked all ratios to assess whether the treated unit’s result was more extreme than those of placebo units, generating a *p*-value that reflects the likelihood of observing such an effect by chance if the policy had no effect. A smaller *p*-value indicates stronger evidence of a policy-outcome relationship.

To understand what types of firearm injuries are contributing to any relationships observed, we re-performed all analyses on [1] rates of nonfatal firearm assault [2], firearm homicide [3], rates of other-directed firearm violence (combining firearm assault and homicide) [4], rates of nonfatal firearm self-harm [5], rates of firearm suicide, and [6] rates of self-directed firearm violence (combining firearm self-harm and suicide).

## Results

### Descriptive findings

Table 2 compares covariates between treated and control states including: firearm ownership and policy; behavioral health; sociodemographic, economic, and political variables; incarceration prevalence; and COVID-19-related attributes. Significant differences across covariates demonstrate that states without ERPOs are a poor proxy for the counterfactual of states with ERPOs, supporting the use of synthetic control modeling.

**Table 2:** Descriptive summary of state-level covariates by treatment status

Firearm ownership & policy covariates	Treated states^1^	Donor pool states^2^	T-test
Proportion of state-months that prohibit firearm possession by those involuntarily committed for inpatient mental health treatment	96.1%	50.6%	< 0.001
Proportion of state-months that prohibit firearm possession by those involuntarily committed for outpatient mental health treatment	70.4%	16.0%	< 0.001
Proportion of state-months that prohibit firearm possession by people who have received treatment for alcoholism that exceeds a state-defined threshold	0.0%	20.0%	< 0.001
Proportion of state-months that prohibit firearm possession by people who have received treatment for alcohol-related problems that exceeds a state-defined threshold	14.3%	12.0%	< 0.001
Proportion of state-months that prohibit firearm possession by people who have been convicted of a drug-related misdemeanor	42.9%	8.0%	< 0.001
Proportion of state-months that prohibit firearm possession by people who have been convicted of a felony	85.7%	72.0%	< 0.001
Proportion of state-months that prohibit firearm possession by people who have committed violent misdemeanors punishable by more than one year of imprisonment	57.1%	12.0%	< 0.001
Proportion of state-months without a ‘stand your ground’ law	85.7%	18.7%	< 0.001
Estimated proportion of state residents who own a firearm	43.6%	59.0%	< 0.001
	**Treated** **states** ^**1**^	**Donor pool states** ^**2**^	**T-test**
**Behavioral health covariates**			
Mean proportion of state residents in the upper 80th percentile of self-reported alcohol consumption	31.8%	28.9%	< 0.001
Mean proportion of state residents with 14 or more “not good” mental health days in the last month	12.7%	14.1%	< 0.001
**Demographic covariates**			
Mean proportion of state residents under 18	20.7%	23.1%	< 0.001
Mean proportion of state residents between 18–34	20.5%	20.1%	< 0.001
Mean proportion of state residents 65 or older	17.4%	16.3%	< 0.001
Mean state population density	513.34 per sq. mile	109.34 per sq. mile	< 0.001
Mean proportion of state residents that identify as White	75.7%	70.5%	< 0.001
Mean proportion of state residents with at least a Bachelor’s degree	36.4%	29.3%	< 0.001
Mean proportion of state residents that were born outside of the US	14.4%	5.9%	< 0.001
Mean proportion of state residents that identify as a veteran	6.7%	6.7%	< 0.001
**Economic covariates**			
Mean state income	$96,878.39	$78,564.84	< 0.001
Mean proportion of state residents that are unemployed	5.7%	5.3%	< 0.001
Proportion of wealth held by a state’s wealthiest decile	49.2%	44.7%	< 0.001
Mean proportion of state residents living in poverty	9.8%	7.8%	< 0.001
Mean proportion of state residents that are uninsured	6.5%	8.8%	< 0.001
	**Treated** **states** ^**1**^	**Donor pool states** ^**2**^	**T-test**
**Incarceration prevalence covariates**			
Mean state incarceration rate	264.7 per 100,000	388.8 per 100,000	< 0.001
**Political covariates**			
Mean score of Republican Control of legislative houses and executive branch (minimum 0, maximum 3)	0.2	0.9	< 0.001
**COVID-19 covariates**			
Mean state COVID-19 death rate	71.05 per 100,000	74.69 per 100,000	< 0.001
Mean number of weeks spent in COVID-19 lockdowns	8.6	5.7	< 0.001

Treated states include: CA, DE, FL, IL, MA, MD, OR, RI, WA, VT

Donor pool states used to derive a synthetic control for each treated state include: AL, AK, AZ, AR, GA, IA, IN, KS, KY, LA, MI, MN, MS, MO, MT, NE, NC, OH, OK, PA, SC, TN, UT, WV, WI

### Analytic findings

The models constructed for six of the ten states (Florida, Massachusetts, Oregon, Rhode Island, Vermont, Washington) offered strong pre-policy period fits. Our model for four treatment states (California, Delaware, Illinois, Maryland) offered a poor pre-policy period fit (Supplement C), making these states’ results uninterpretable. Model specifications including donor unit weights (Supplement D) and covariate values for treated states and their synthetic controls (Supplement E) are similarly appended.

Descriptive trends in ERPO petition volume and firearm violence outcomes of interest for states with interpretable findings are offered in Table 3. Estimates for Florida, Massachusetts, Oregon, Rhode Island, Vermont, and Washington suggest very different impacts across states and over post-implementation years (Table 4). In Florida, Oregon, Washington, and Vermont, there was no observed relationship between ERPO enactment and firearm violence incidents. Contrastingly, in Massachusetts and Rhode Island, ERPO enactment was associated with statistically significant reductions (*p*-value: 0.04 for both) in firearm violence incidents. Relative to the rates expected in each year per ‘synthetic Massachusetts’, the Massachusetts model suggests 2.60 [95% CI: -1.928, -3.275 ] fewer incidents per 1,000,000 residents occurred in implementation year one (-4.26%), 4.52 [95% CI: -3.036, -6.006 ] fewer incidents per 1,000,000 residents in year two (-8.45%), 7.98 [95% CI: -6.892, -9.080 ] fewer per 1,000,000 in year three (-13.42%), and 7.89 [95% CI: -7.062, -8.722 ] fewer per 1,000,000 in year four (17.69%).

**Table 3:**
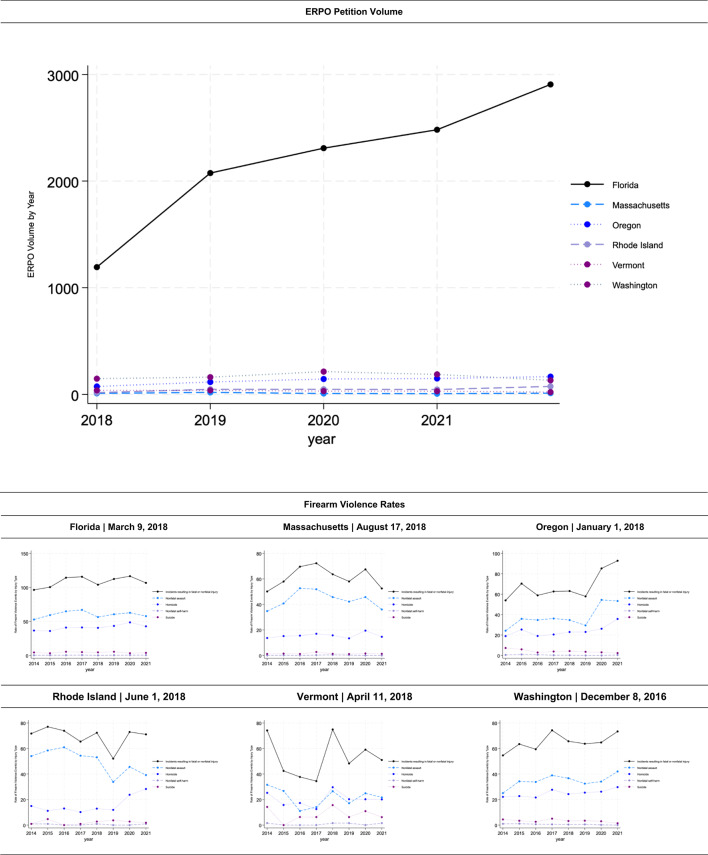
Descriptive trends of ERPO petitions & firearm violence events resulting in death or injury for treated States with pre-period fit

**Table 4:**
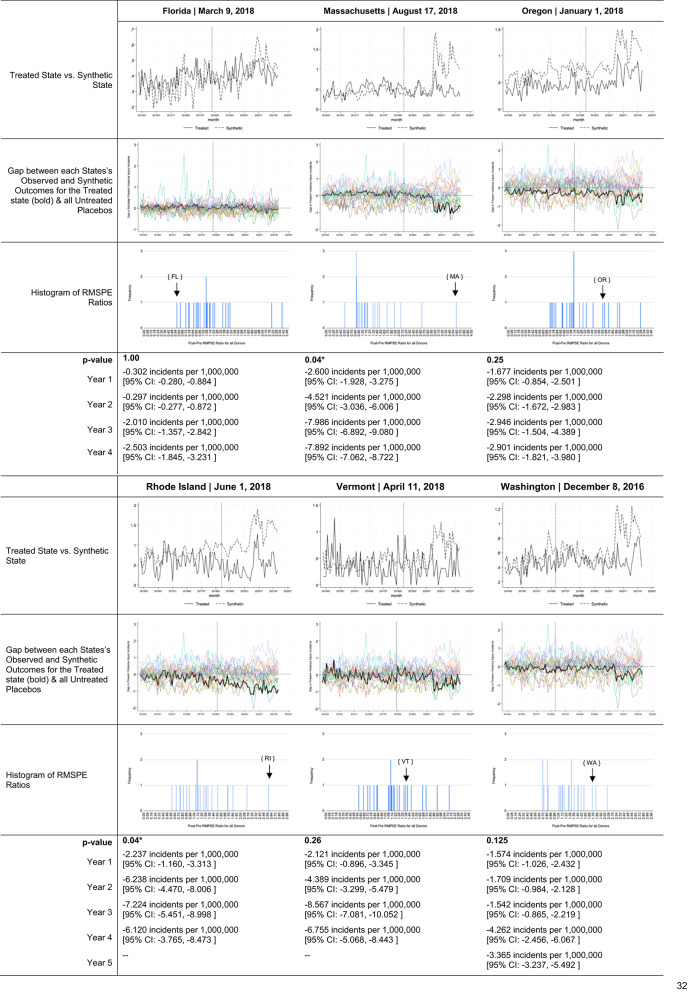
Estimates of ERPO impact on firearm violence events resulting in death or injury for treated states with pre-period fit

**Table 5:**
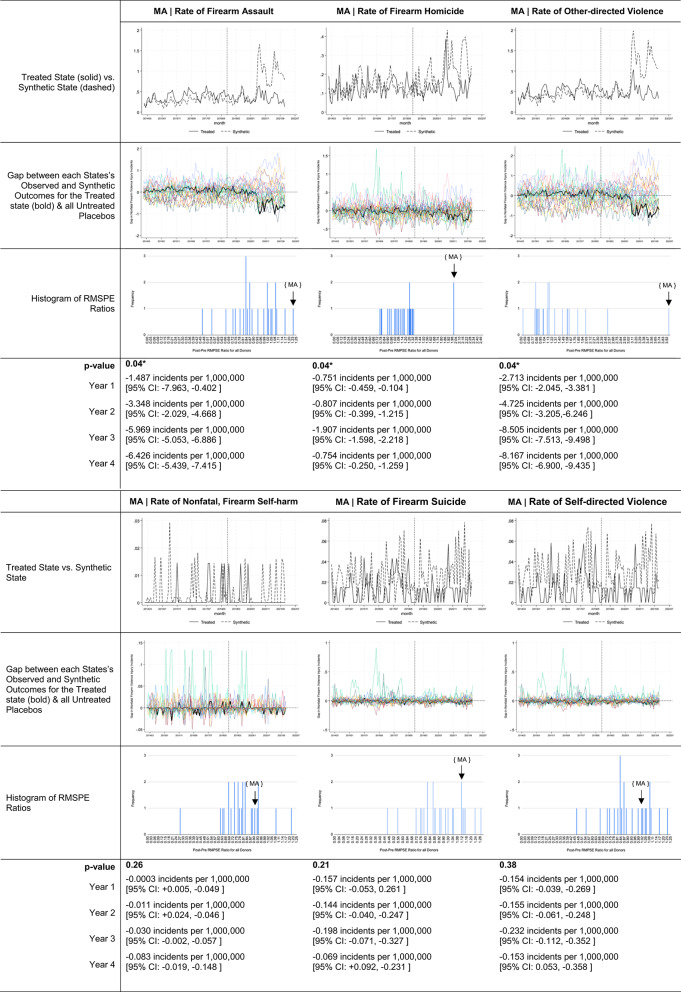
Estimates of ERPO impact on specific types of firearm violence events for Massachusetts

**Table 6:**
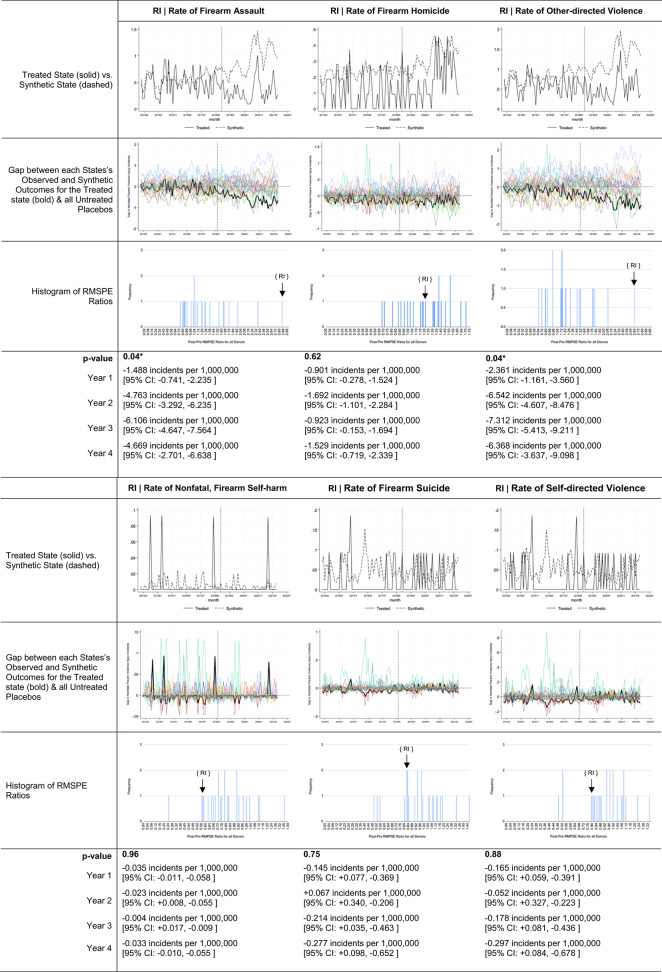
Estimates of ERPO impact on specific types of firearm violence events for Rhode Island

## Discussion

This quasi-experimental study suggests that not all ERPO policies are created and implemented equally. ERPO enactment in Rhode Island and Massachusetts resulted in significant reductions in firearm violence incidents yielding injury or death. Notably, when stratifying by intent, the significant post-enactment reductions in firearm violence in both Rhode Island and Massachusetts were primarily driven by decreases in firearm assault. Prior literature has largely demonstrated an association between ERPOs and reductions in firearm suicide, and ERPO petitions are more commonly used to prevent self-directed harm than interpersonal violence [11, 29–31]. Our findings suggest a potential underrecognized, cross-intent impacts of ERPOs on violent crime.

Contrastingly, under our model conditions Florida’s, Oregon’s, Vermont’s, and Washington’s ERPO policies appear to have no impact on injuries and deaths due to firearm violence, despite variations in ERPO specifications. Thus, it is also possible that ERPOs are not associated with population-level reductions in firearm violence incidents and deaths. Similar to other studies examining population-level impact, this study may reflect the underlying principle that ERPOs are issued to a small subset of individuals, and the aggregate impact of ERPOs on state-level firearm violence incident rates is difficult to detect [16, 32]. The absence of observed effect in most states may reflect lack of knowledge of ERPOs, barriers to petitioning, and challenges in firearm removal [33–36]. 

It is difficult to empirically confirm potential mechanisms leading to reductions in firearm violence incidents given related literature on ERPO implementation is limited across states with statistically significant results. With respect to Rhode Island, grey literature suggests that in the first year of Rhode Island’s ERPO implementation (2018), large metropolitan counties (Cranston, Warwick) and smaller rural counties (Central Falls, Westerly) all reported at least one petition, demonstrating broad reach throughout the state [37]. After the 2014–2021 period analyzed here, Rhode Island received $1.2 million from the BSCA of 2022 for expanding ERPO implementation and crisis board development, though this was not distributed until 2023. In addition, Massachusetts has implemented a data dashboard that tracks the number of petitions filed per month, their outcomes, and respondent characteristics, demonstrating a robust infrastructure to support ERPO awareness, use, and data collection [38]. 

The magnitude of the relationship in both Massachusetts and Rhode Island generally increased overtime, peaking with the onset of the COVID-19 pandemic in 2020. This may also suggest there was an increased need for and uptake of ERPO petitions during that period, which in part may be due to an increase in firearm purchasing during this period of time [37, 39]. In Rhode Island, state capacity for implementing ERPOs effectively may have improved over time suggested by year-to-year increases in petition volumes, in turn improving ERPO’s protective potential. This could reflect a growing awareness of and investment in ERPO implementation. Furthermore, larger, national investments through the 2022 Bipartisan Safer Communities Act (BSCA), which included $750 million in federal grant funding to bolster state ERPO implementation, also demonstrate an expanding landscape of nationwide investment.

Importantly, Rhode Island’s and Massachusetts’ ERPO laws were each associated with statistically significant reductions in firearm violence incidents, despite relatively small petition volumes (109 and 44 total during the study period, respectively). This suggests that the mechanisms contributing to reduced firearm violence may extend beyond the number of petitions filed and/or relate to co-occurring firearm violence prevention efforts. For instance, our models did not adjust for concurrent community violence intervention (CVI) investments during the study period. Massachusetts appropriated an additional $25 million for CVI efforts, while Rhode Island received $1.8 million from the U.S. Department of Justice and $2.6 million from the American Rescue Plan Act for CVI initiatives [40–42]. Thus, complementary policy and programmatic efforts that were unable to be accounted for in our model may therefore contribute in part or in whole to the observed reductions noted in our analysis.

To our knowledge, this study is the first to include nonfatal firearm violence incidents in statewide population level analyses of ERPOs. Data limitations have undermined the research community’s ability to explore these events to the same extent as fatal violence incidents [43]. Findings suggest ERPO adoption may protect against firearm assault incidents in particular. The greater prevalence of nonfatal events likely supported the detection of an effect, whereas other studies including only more rare outcomes (e.g., completed suicides, mass shootings) have not discerned significant relationships. For example, Pear et al. acknowledged this limitation when looking at ERPO’s impact on firearm suicide and nonfatal firearm assault rates in San Diego County, California [16]. In the case of firearm suicide, most people who experience active suicidal ideation do not die by suicide. It is almost impossible to know if someone who had an ERPO petition filed against them would have gone on to commit a violent act. It is also possible that ERPOs in their current form and scale of implementation may not be sufficient to reduce population-level suicide rates, even if they prevent suicide in high-risk individuals. This is consistent with studies demonstrating ERPOs can prevent suicide among respondents while still yielding minimal or null effects in community-level analyses [14]. 

A key strength of this analysis is the ability to estimate state-specific ERPO impacts over time. It is important to note that communities within these states may experience ERPO policies differently. Furthermore, the effectiveness of ERPO implementation may depend less on petition volume alone and more on factors such as petitioner awareness, broad regional uptake, appropriate application of the law, judicial system preparedness, and robust data collection and monitoring. Efforts to strengthen implementation should therefore include increasing awareness across all classes of petitioners, ensuring judicial officials are adequately trained, improving the identification of individuals at risk, and establishing comprehensive data systems with real-time feedback to support continuous improvement.

In applying quasi-experimental methods, our findings are useful for the policymaking community and to practitioners involved in ERPO implementation. For states without ERPOs, stakeholders can model their policy and implementation approaches after states achieving significant reductions in firearm injury and death incidents, prioritizing continued evaluation and resource allocation. For states with ERPO provisions, policymakers and stakeholders can identify differences between their state’s existing ERPOs and those yielding significant reductions and modify their approaches accordingly.

## Limitations

Our study has limitations. First, as with other evaluations of policy impacts on firearm violence, it is not possible to determine whether firearm violence incidents occurring during the pre-implementation period in each state would have been eligible for an ERPO had the law been in place. This limitation makes causal attribution challenging, as ERPOs are designed to mitigate imminent, individual-level risk rather than population-level violence broadly. Descriptive studies of ERPO petitions indicate that many respondents exhibit documented warning signs, such as prior threats, violent ideation, suicidal behavior, or escalating interpersonal conflict, suggesting that some incidents of interpersonal or self-directed firearm violence could have met ERPO criteria had risk been identified and a petition filed. However, ERPO eligibility is unlikely to extend to all forms of firearm injury, including unintentional shootings or incidents arising from sudden escalation (e.g., road rage or group-involved violence), underscoring the importance of interpreting observed population-level associations cautiously [44–46]. 

Second, while GVA is one of the few data sources that compiles nonfatal firearm violence incident data, it sources its data in part from media outlets which may omit nonfatal firearm incidents that are not reported on. This potentially dilutes our estimates. Additionally, while only contributing to a minority of incidents, law-enforcement involved incidents and self-defensive firearm events were not excluded.

Third, the recency of the ERPO passage in many states created challenges. The short post-implementation period resulted in the omission of six states. Even for included states, the impact estimates changed considerably with each additional year. Therefore, as the post-policy implementation periods across states lengthen, replication of these analyses would lend valuable insights on ERPO impacts over time. Additionally, earlier ERPO laws have since been updated, and more recent statutes may have incorporated lessons from these initial, imperfect versions, potentially enhancing their impact on nonfatal firearm injuries.

Fourth, as in all quasi-experimental approaches, there is no definitive way to empirically confirm all potential confounders (or time varying differences other than the exposure of interest) are at play. To mitigate the risk of misattributing changes in firearm violence rates to ERPOs when other contributors could be present, estimates are only offered in instances where strong pre-period fit is exhibited, and covariates were carefully selected to address foreseeable confounders (e.g., COVID-19 burden, other firearm safety policies. Relatedly, our models do not account for concurrent community violence intervention (CVI) investments during the study period. Both Massachusetts and Rhode Island substantially increased funding for CVI initiatives [40–42]. Given that ERPOs are issued to a relatively small number of individuals, the magnitude of the observed population-level effects is unlikely to reflect a direct, one-to-one relationship between ERPO petitions and reduced assaults. It is plausible that the observed reductions are due in whole or in part to policy and programmatic interventions occurring contemporaneously with ERPO implementation.

Fifth, although we exclude states without a significant increase in petition volume post-enactment, our models lack detailed measures of implementation over time. Differences in ERPO impact estimates may reflect such gaps. Oregon, for example, sees more family-initiated ERPOs, which are less likely to be granted due to inexperience with legal forms and lower perceived credibility compared to law enforcement-initiated petitions [29]. This potentially contributed to Oregon’s modest results. For these and other reasons, high petition numbers may not translate to policy impact across states. Additionally, in states such as Oregon and Washington, a substantial proportion of ERPOs are filed in response to self-harm risk, which may have limited impact on reducing interpersonal firearm injuries; however, many petitions also cite combined threats to self and others [29–31, 47]. Qualitative research is needed to further explore implementation barriers and facilitators.

Sixth, performing a state-level analysis collapses considerable county-level variation. In response to ERPOs, many local governments have declared themselves Second Amendment (2 A) Sanctuaries wherein restrictive firearm policies are perceived as unconstitutional are not enforced [48, 49]. In a study of Colorado ERPOs, petitions filed in Second Amendment (2 A) sanctuary jurisdictions were less likely to be initiated by law enforcement and less likely to result in an emergency order being granted, although ERPOs were still issued in cases involving threats of self-harm, interpersonal violence, or mass violence [50]. Consequently, some individuals eligible for ERPOs in these jurisdictions may not receive them, which could dilute the overall impact of the intervention and contribute to a negligible policy effect. For these and other reasons, the estimates likely miss considerable within-state variation of ERPO impact. Future county-level quasi-experimental analyses may accordingly add value.

Seventh, while we controlled for other laws shaping firearm access within states, we did not control for those held by surrounding states. Surrounding states’ firearm laws impact illegal exchange of firearms [51]. The Northeast census region holds more stringent firearm laws compared to the rest of the country, which may contribute to regional trends in reduction in firearm violence incidents.

Lastly, our analysis precluded us from stratifying by victim identity to consider differences in ERPO effectiveness across demographic sub-groups. Prior works note ERPO respondents are predominantly white [52], and the equity implications of this trend are not yet fully understood. Economic disinvestment, discriminatory policies, and systemic racism have led to interpersonal firearm violence disproportionately impacting individuals from minoritized racial and ethnic backgrounds, specifically Black adolescents and young adults [53, 54]. Similarly, self-directed violence disproportionately impacts rural communities [1]. 

## Conclusions

In this synthetic control analysis, we found that the impact of ERPOs on fatal and nonfatal firearm violence incidents varies notably between states. While some states experienced statistically significant decreases following ERPO implementation (Massachusetts, Rhode Island), others saw no change (Florida, Oregon, Vermont, Washington). Where significant declines were observed, effect sizes suggest factors other than ERPO policies may be contributing. Not all ERPO policies are created or implemented equally. Under certain enactment and implementation conditions, ERPO policies may protect against nonfatal firearm assault incidents in particular.

## Supplementary Information

Supplementary Material 1.

## Data Availability

All data used for the in this work is publicly available and specified in the methods section.

## Abbreviations

ERPOs: Extreme Risk Protection Orders
COVID-19: Coronavirus Disease 2019, or SARS-CoV-2/Severe Acute Respiratory Syndrome Coronavirus 2
BRFSS: Behavioral Risk Factor Surveillance System
RMSPE: Root mean square prediction error
BSCA: 2022 Bipartisan Safer Communities Act
CVI: Community violence interventions
2A: Second amendment
