# Supplementary material for: Extreme risk protection orders and firearm violence: a synthetic control analysis
Source: Inj Epidemiol. 2026 Feb 16;13:22. doi: 10.1186/s40621-026-00663-8 (PMC13014952; doi:10.1186/s40621-026-00663-8)

**Supplement A**

Paired t*-*tests were conducted at the state level to assess whether there was a statistically significant change (alpha = 0.05) in the annual number of ERPO petitions filed after law implementation compared with the pre-implementation period, during which the annual petition volume was zero. This approach enabled identification of states demonstrating sustained, nonzero ERPO activity following enactment. Those with statistically significant increases in annual petition counts. This method provides a straightforward and reproducible approach for quantifying implementation intensity across states in quasi-experimental analyses.

**States With and Without Statistically Significant Post-Enactment Increases in ERPO Petitions Using 2-Sided t-test of Significance**

| **States with Statistically Significant ERPO Petition Increases [State(FIPS)]** | **States without Statistically Significant ERPO Petition Increases [State(Fips)]** |
| --- | --- |
| California (6)  Connecticut (9)  Delaware (10)  Florida (12)  Illinois (17)  Maryland (24)  Massachusetts (25)  Nevada (32)  New Jersey (34)  Oregon (41)  Rhode Island (44)  Vermont (50)  Washington (53) | Colorado (8)  Hawaii (15)  New Mexico (35)  New York (36)  Virginia (51) |

STATA 18.5 code:

use "/file_path/ERPOThreshold.dta" //Paired T-test Comparison for change in ERPOS filed before and after enacted

rename erposfiled erposfiled_after //generate an after ERPO category

generate erposfiled_before = 0 //generate a before ERPO category

drop if erpoactive != 1 //consider only state-years with ERPOS

sort fipscode year //sort by fips and year to allow 'by' command to run

by fipscode: ttest erposfiled_before = erposfiled_after //generates a paired t-test for each state's data so we can identify states that experienced significant changes in petition numbers and those that did not

//Significant for 2-sided test: 6, 9, 10, 12, 17, 24, 25, 32, 34, 41, 44, 50, 53

//Not Significant for 2-sided test: 8, 15, 35, 36, 51

**Supplement B**

Table S2. Data Source Table

| **Data Source** | **Variable** | **Year(s)** | **Variable for Linkage** | **Value** | | **Type** | | **Notes** | | |
| --- | --- | --- | --- | --- | --- | --- | --- | --- | --- | --- |
| **Explanatory Data** | | | | | | | | | | |
| National Extreme Risk Protection Order Resource Center (ERPO.org) | Presence of an ERPO Policy | 2014-2021 | State FIPS | Categorical | | Discrete | | Represents when the policy was enacted | | |
| **Outcome Data** | | | | | | | | | | |
| Gun Violence Archives | Firearm Violence Incidents | 2014-2021 | State FIPS | Numeric | | Continuous | | The Gun Violence Archives defines a firearm violence incident as those involving firearm-related injuries or deaths, including assaults/homicide, self-directed violence/suicide, mass shootings, and unintentional injuries. | | |
| **Covariates** | | | | | | | | | | |
| Centers for Disease Control and prevention Web-based Injury Statistics Query and Reporting System | Firearm Ownership | 2014-2021 | State FIPS | Numeric | | Discrete | | A proxy measure of firearm ownership was derived by calculating the proportion of suicides committed with a firearm relative to the total suicides in a given state and year. | | |
| Siegel Firearm State Law Database | High-Risk Restrictive Firearm Legislation | 2014-2020 | State FIPS | Numeric | | Discrete | | Number of total firearm restrictive laws for each individual state-year   - Short term in - Short term out - Alcoholism - Felony - Violent Partial - Alc Treatment - Nosyg - Drug Misdemeanor | | |
| **Data Source** | **Variable** | **Year(s)** | **Variable for Linkage** | **Value** | | **Type** | | **Notes** | | |
| National Council of State Legislators | Republican Political Control | 2014-2021 | State FIPS | Numeric | | Discrete | | Number of chambers controlled by the Republican Party for each state year. Chambers included State House of Representatives, State Senate, and Governor. | | |
| Federal Bureau of Investigation Uniform Crime Reporting System | Incarceration Rate | 2014-2021 | State FIPS | Numeric | | Continuous | | Incarcerated Persons Per Capita | | |
| World Inequality Database | Share of Income for Top 10% | 2014-2018 | State FIPS | Numeric | | Continuous | | A measure of income inequality included in the World Inequality Database | | |
| Behavioral Risk Factor Surveillance System (BRFSS) | Rate of Alcohol Use | 2014-2021 | State FIPS | Numeric | Categorical | | | Percent of state residents in the upper quintile of alcohol consumption nationally for each year | | |
|  | Rate of Poor Mental health Days | 2014-2021 | State FIPS | Numeric | | Categorical | | Percent of state population with at least 14 not good mental health days in the last 30 for each state year | | |
| Integrated Public Use Microdata Series (IPUMS) for the United States Census Bureau American Community Survey (ACS) | Median Household Income | 2014-2021 | State FIPS | Numeric | | Continuous | | Adjusted for year-to-year inflation rates | | |
|  | State population density | 2014-2021 | State FIPS | Numeric | | Continuous | |  | | |
| **Data Source** | **Variable** | **Year(s)** | **Variable for Linkage** | **Value** | | **Type** | | **Notes** | | |
|  | Percent of state population unemployed | 2014-2021 | State FIPS | Numeric | | | Continuous | | Civilian population in labor force, 16 years and older |  |
|  | Percent state population Age Under 18 | 2014-2021 | State FIPS | Numeric | | | Continuous | |  |  |
|  | Percent state population Age 18 to 34 | 2014-2021 | State FIPS | Numeric | | | Continuous | |  |  |
|  | Percent state population Age 35 to 64 | 2014-2021 | State FIPS | Numeric | | | Continuous | |  |  |
|  | Percent state population Age 65 and Over | 2014-2021 | State FIPS | Numeric | | | Continuous | |  |  |
|  | Percent state population bachelor’s degree | 2014-2021 | State FIPS | Numeric | | | Continuous | |  |  |
|  | Percent state population Non-Hispanic White | 2014-2021 | State FIPS | Numeric | | | Continuous | |  |  |
|  | Percent of state population Living in Poverty | 2014-2021 | State FIPS | Numeric | | | Continuous | | At or below the poverty rate for the specified year |  |
|  | Percent of state population non-US Citizens | 2014-2021 | State FIPS | Numeric | | | Continuous | |  |  |
|  | Percent of state population uninsured | 2014-2021 | State FIPS | Numeric | | | Continuous | |  |  |
|  | Percent of state population that are veterans | 2014-2021 | State FIPS | Numeric | | | Continuous | | 18 years and older |  |
| Everytown for Gun Safety | Numbers of ERPOs Petitioned | Varied | State FIPS | Numeric | | Discrete | | Year to year variations in number of ERPOs implemented | | |
| Ballotpedia.com | Active Lockdown | Varied | State FIPS | Categorical | | Discrete | | State to state variations in duration of lockdown | | |
| **Data Source** | **Variable** | **Year(s)** | **Variable for Linkage** | **Value** | | **Type** | | **Notes** | | |
| CDC COVID Data Tracker | COVID Deaths | 2020-2021 | State FIPS | Numeric | | Continuous | | State-Month | | |

**Supplement C**

Figure S1. Synthetic Control Outputs for of ERPO provisions and effective dates for Treated states with poor pre-period fit

| **California \| January 1, 2016** | **Delaware \| December 27, 2018** |
| --- | --- |
| 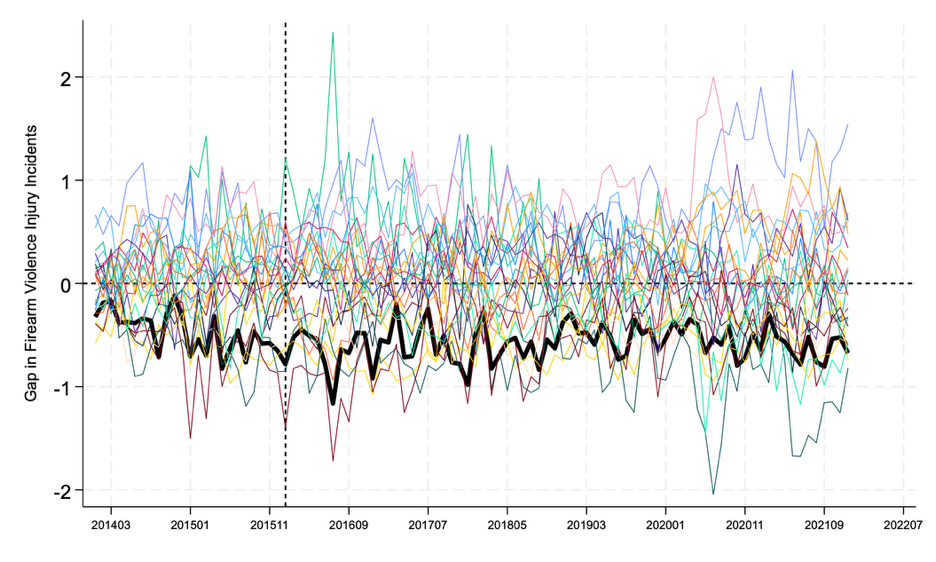 | 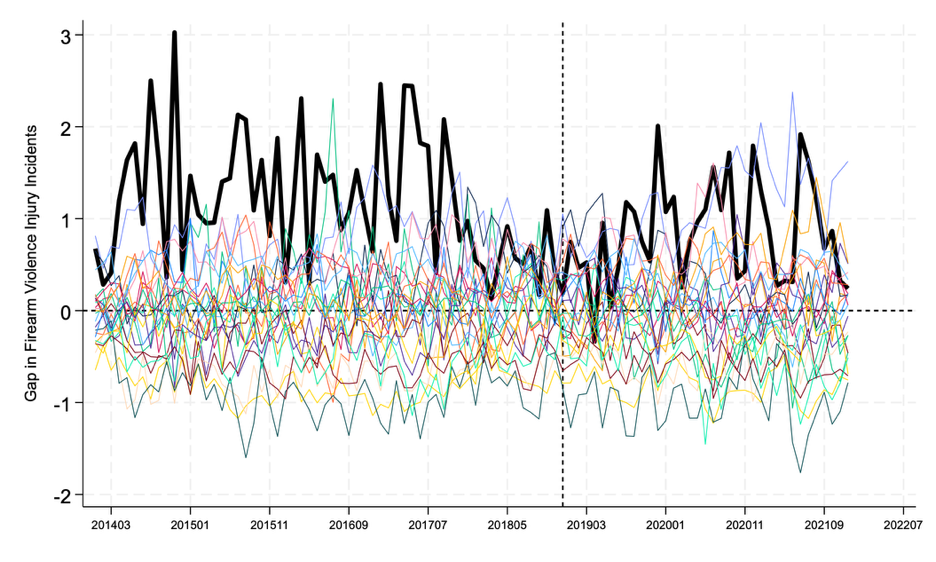 |
| **Illinois \| January 1, 2019** | **Maryland \| October 1, 2018** |
| 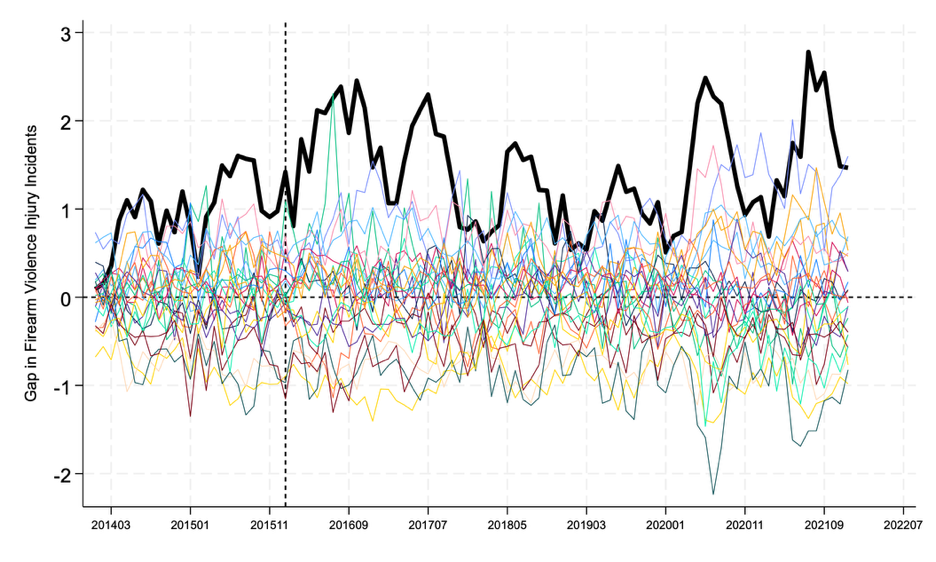 | 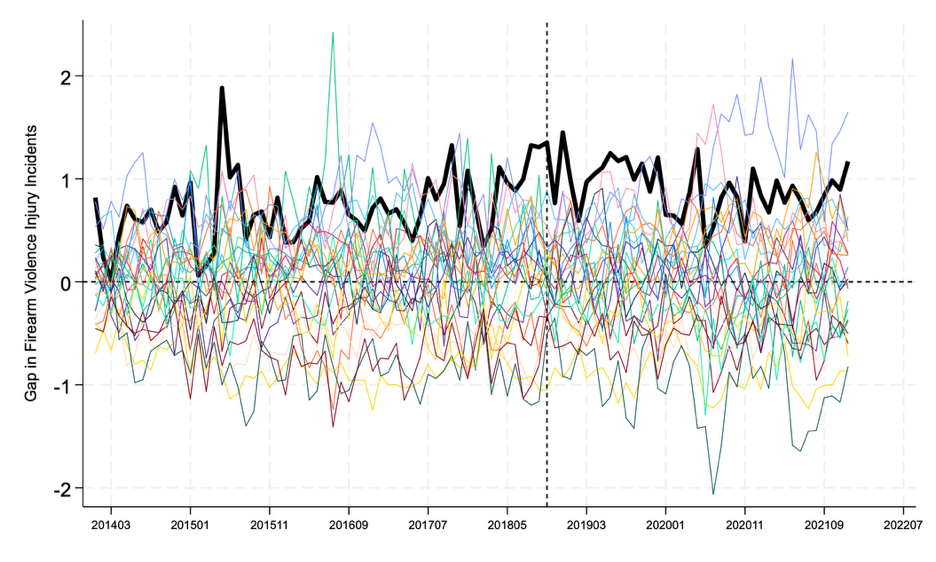 |

**Supplement D**

Table S1. Donor Unit Weights applied to generate each Synthetic Treatment State

| **California** | **Delaware** | **Florida** | **Illinois** | **Maryland** |
| --- | --- | --- | --- | --- |
| Alaska (0.348) | Arizona (0.180) | Arizona (0.613) | Alaska (0.119) | Alaska (0.108) |
| Georgia (0.23) | Minnesota (0.270) | Michigan (0.052) | Arizona (0.098) | Georgia (0.291) |
| Minnesota (0.35) | Montana (0.300) | Montana (0.190) | Georgia (0.057) | Minnesota (0.595) |
| Pennsylvania (0.363) | Pennsylvania (0.246) | Pennsylvania (0.101) | Minnesota (0.301) | Pennsylvania (0.006) |
| Utah (0.024) | West Virginia (0.135) | West Virginia (0.044) | Pennsylvania (0.41) |  |
|  |  |  | Utah (0.015) |  |
|  |  |  |  |  |
| **Massachusetts** | **Oregon** | **Rhode Island** | **Vermont** | **Washington** |
| Michigan (0.122) | Alaska (0.086) | Alaska (0.031) | Iowa (0.099) | Alaska (0.145) |
| Minnesota (0.463) | Arizona (0.262) | Minnesota (0.060) | Minnesota (0.406) | Arizona (0.310) |
| Utah (0.415) | Minnesota (0.271) | Pennsylvania (0.486) | Montana (0.495) | Michigan (0.028) |
|  | Pennsylvania (0.169) | Utah (0.217) |  | Minnesota (0.468) |
|  | West Virginia (0.069) | Wisconsin (0.207) |  | Utah (0.049) |
|  | Wisconsin (0.143) |  |  |  |
|  |  |  |  |  |

**Supplement E**

Table S3. Predictor Values for Delaware and Synthetic California

| **Covariates** | **California** | **Synthetic California** |
| --- | --- | --- |
| **Firearm Ownership & Policy Covariates** |  |  |
| Proportion of state-months that prohibit firearm possession by those involuntarily committed for inpatient mental health treatment | 100% | 42.2% |
| Proportion of state-months that prohibit firearm possession by those involuntarily committed for outpatient mental health treatment | 0.0% | 0.0% |
| Proportion of state-months that prohibit firearm possession by people who have received treatment for alcoholism that exceeds a state-defined threshold | 100% | 0.0% |
| Proportion of state-months that prohibit firearm possession by people who have received treatment for alcohol-related problems that exceeds a state-defined threshold | 0.0% | 3.5% |
| Proportion of state-months that prohibit firearm possession by people who have been convicted of a drug-related misdemeanor | 0.0% | 39.8% |
| Proportion of state-months that prohibit firearm possession by people who have been convicted of a felony | 100.0% | 63.7% |
| Proportion of state-months that prohibit firearm possession by people who have committed violent misdemeanors punishable by more than one year of imprisonment | 100.0% | 39.8% |
| Proportion of state-months without a ‘stand your ground’ law | 100.0% | 3.5% |
| Estimated proportion of state residents who own a firearm | 36.9% | 57.1% |
|  |  |  |
| **Behavioral Health Covariates** |  |  |
| Mean proportion of state residents in the upper 80^th^ percentile of self-reported alcohol consumption | 33.9% | 32.5% |
| Mean proportion of state residents with 14 or more “not good” mental health days in the last month | 11.6% | 12.1% |
|  |  |  |
| **Demographic Covariates** |  |  |
| Mean proportion of state residents under 18 | 22.5% | 23.3% |
| Mean proportion of state residents between 18-34 | 22.5% | 21.5% |
| Mean proportion of state residents 65 or older | 14.0% | 14.3% |
| Mean state population density | 252.23 per sq. mile | 149.47 per sq. mile |
| Mean proportion of state residents that identify as White | 56.8% | 69.4% |
| Mean proportion of state residents with at least a Bachelor’s degree | 33.8% | 31.1% |
| Mean proportion of state residents that were born outside of the US | 26.9% | 8.0% |
| Mean proportion of state residents that identify as a veteran | 0.05% | 0.04% |
| **Economic Covariates** |  |  |
| Mean state income | $104,074.30 | $86,932.54 |
| Mean proportion of state residents that are unemployed | 6.7% | 6.2% |
| Proportion of wealth held by a state’s wealthiest decile | 52.0% | 42.5% |
| Mean proportion of state residents living in poverty | 9.9% | 8.6% |
| Mean proportion of state residents that are uninsured | 8.1% | 10.4% |
|  |  |  |
| **Incarceration Prevalence Covariates** |  |  |
| Mean state incarceration rate | 310.24 per 100,000 | 343.74 per 100,000 |
| **Political Covariates** |  |  |
| Mean score of Republican Control of legislative houses and executive branch (minimum 0, maximum 3) | 0 | 0.82 |
|  |  |  |
| **COVID-19 Covariates** |  |  |
| Mean state COVID-19 death rate | 68.8 per 100,000 | 91.2 per 100,000 |
| Mean number of weeks spent in COVID-19 lockdowns | 23.0 | 8.0 |
|  |  |  |

Table S4. Predictor Values for Delaware and Synthetic Delaware

| **Covariates** | **Delaware** | **Synthetic Delaware** |
| --- | --- | --- |
| **Firearm Ownership & Policy Covariates** |  |  |
| Proportion of state-months that prohibit firearm possession by those involuntarily committed for inpatient mental health treatment | 100% | 89.7% |
| Proportion of state-months that prohibit firearm possession by those involuntarily committed for outpatient mental health treatment | 100% | 31.5% |
| Proportion of state-months that prohibit firearm possession by people who have received treatment for alcoholism that exceeds a state-defined threshold | 0.0% | 13.5% |
| Proportion of state-months that prohibit firearm possession by people who have received treatment for alcohol-related problems that exceeds a state-defined threshold | 0.0% | 33.6% |
| Proportion of state-months that prohibit firearm possession by people who have been convicted of a drug-related misdemeanor | 100.0% | 58.2% |
| Proportion of state-months that prohibit firearm possession by people who have been convicted of a felony | 100.0% | 65.1% |
| Proportion of state-months that prohibit firearm possession by people who have committed violent misdemeanors punishable by more than one year of imprisonment | 100.0% | 71.7% |
| Proportion of state-months without a ‘stand your ground’ law | 100.0% | 33.6% |
| Estimated proportion of state residents who own a firearm | 48.2% | 53.2% |
|  |  |  |
| **Behavioral Health Covariates** |  |  |
| Mean proportion of state residents in the upper 80^th^ percentile of self-reported alcohol consumption | 31.7% | 31.7% |
| Mean proportion of state residents with 14 or more “not good” mental health days in the last month | 12.0% | 12.4% |
|  |  |  |
| **Demographic Covariates** |  |  |
| Mean proportion of state residents under 18 | 21.2% | 22.1% |
| Mean proportion of state residents between 18-34 | 18.8% | 19.0% |
| Mean proportion of state residents 65 or older | 18.3% | 17.2% |
| Mean state population density | 494.46 per sq. mile | 116.12 per sq. mile |
| Mean proportion of state residents that identify as White | 67.6% | 82.4% |
| Mean proportion of state residents with at least a Bachelor’s degree | 32.1% | 31.4% |
| Mean proportion of state residents that were born outside of the US | 9.5% | 7.3% |
| Mean proportion of state residents that identify as a veteran | 0.1% | 0.1% |
| **Economic Covariates** |  |  |
| Mean state income | $86,637.67 | $81,283.21 |
| Mean proportion of state residents that are unemployed | 5.6% | 5.2% |
| Proportion of wealth held by a state’s wealthiest decile | 45.2% | 45.9% |
| Mean proportion of state residents living in poverty | 8.3% | 8.6% |
| Mean proportion of state residents that are uninsured | 6.1% | 6.% |
|  |  |  |
| **Incarceration Prevalence Covariates** |  |  |
| Mean state incarceration rate | 388.82 per 100,000 | 335.24 per 100,000 |
| **Political Covariates** |  |  |
| Mean score of Republican Control of legislative houses and executive branch (minimum 0, maximum 3) | 0 | 0.65 |
|  |  |  |
| **COVID-19 Covariates** |  |  |
| Mean state COVID-19 death rate | 66.7 per 100,000 | 85.2 per 100,000 |
| Mean number of weeks spent in COVID-19 lockdowns | 4.2 | 3.0 |
|  |  |  |

Table S5. Predictor Values for Florida and Synthetic Florida

| **Covariates** | **Florida** | **Synthetic Florida** |
| --- | --- | --- |
| **Firearm Ownership & Policy Covariates** |  |  |
| Proportion of state-months that prohibit firearm possession by those involuntarily committed for inpatient mental health treatment | 50.0% | 75.8% |
| Proportion of state-months that prohibit firearm possession by those involuntarily committed for outpatient mental health treatment | 0.0% | 65.7% |
| Proportion of state-months that prohibit firearm possession by people who have received treatment for alcoholism that exceeds a state-defined threshold | 0.0% | 4.4% |
| Proportion of state-months that prohibit firearm possession by people who have received treatment for alcohol-related problems that exceeds a state-defined threshold | 0.0% | 0.0% |
| Proportion of state-months that prohibit firearm possession by people who have been convicted of a drug-related misdemeanor | 0.0% | 10.1% |
| Proportion of state-months that prohibit firearm possession by people who have been convicted of a felony | 100.0% | 70.9% |
| Proportion of state-months that prohibit firearm possession by people who have committed violent misdemeanors punishable by more than one year of imprisonment | 0.0% | 14.5% |
| Proportion of state-months without a ‘stand your ground’ law | 0.0% | 0.0% |
| Estimated proportion of state residents who own a firearm | 53.9% | 57.9% |
|  |  |  |
| **Behavioral Health Covariates** |  |  |
| Mean proportion of state residents in the upper 80^th^ percentile of self-reported alcohol consumption | 33.6% | 31.8% |
| Mean proportion of state residents with 14 or more “not good” mental health days in the last month | 12.2% | 12.7% |
|  |  |  |
| **Demographic Covariates** |  |  |
| Mean proportion of state residents under 18 | 19.9% | 22.4% |
| Mean proportion of state residents between 18-34 | 18.3% | 19.7% |
| Mean proportion of state residents 65 or older | 20.2% | 17.6% |
| Mean state population density | 390.4 per sq. mile | 80.4 per sq. mile |
| Mean proportion of state residents that identify as White | 72.4% | 78.7% |
| Mean proportion of state residents with at least a Bachelor’s degree | 29.8% | 29.81% |
| Mean proportion of state residents that were born outside of the US | 20.69% | 9.68% |
| Mean proportion of state residents that identify as a veteran | 0.08% | 0.07% |
| **Economic Covariates** |  |  |
| Mean state income | $77,917.05 | $77,353.54 |
| Mean proportion of state residents that are unemployed | 5.9% | 5.7% |
| Proportion of wealth held by a state’s wealthiest decile | 60.8% | 47.9% |
| Mean proportion of state residents living in poverty | 10.1% | 10.1% |
| Mean proportion of state residents that are uninsured | 13.3% | 9.7% |
|  |  |  |
| **Incarceration Prevalence Covariates** |  |  |
| Mean state incarceration rate | 449.1 per 100,000 | 481.9 per 100,000 |
| **Political Covariates** |  |  |
| Mean score of Republican Control of legislative houses and executive branch (minimum 0, maximum 3) | 1 | 0.894 |
|  |  |  |
| **COVID-19 Covariates** |  |  |
| Mean state COVID-19 death rate | 67.5 per 100,000 | 86.1 per 100,000 |
| Mean number of weeks spent in COVID-19 lockdowns | 2.1 | 2.9 |
|  |  |  |

Table S6. Predictor Values for Delaware and Synthetic Illinois

| **Covariates** | **Illinois** | **Synthetic Illinois** |
| --- | --- | --- |
| **Firearm Ownership & Policy Covariates** |  |  |
| Proportion of state-months that prohibit firearm possession by those involuntarily committed for inpatient mental health treatment | 100% | 82.4% |
| Proportion of state-months that prohibit firearm possession by those involuntarily committed for outpatient mental health treatment | 100% | 9.8% |
| Proportion of state-months that prohibit firearm possession by people who have received treatment for alcoholism that exceeds a state-defined threshold | 0.0% | 0.0% |
| Proportion of state-months that prohibit firearm possession by people who have received treatment for alcohol-related problems that exceeds a state-defined threshold | 0.0% | 30.1% |
| Proportion of state-months that prohibit firearm possession by people who have been convicted of a drug-related misdemeanor | 100.0% | 71.1% |
| Proportion of state-months that prohibit firearm possession by people who have been convicted of a felony | 100.0% | 59.0% |
| Proportion of state-months that prohibit firearm possession by people who have committed violent misdemeanors punishable by more than one year of imprisonment | 100.0% | 71.1% |
| Proportion of state-months without a ‘stand your ground’ law | 100.0% | 30.1% |
| Estimated proportion of state residents who own a firearm | 38.8% | 51.8% |
|  |  |  |
| **Behavioral Health Covariates** |  |  |
| Mean proportion of state residents in the upper 80^th^ percentile of self-reported alcohol consumption | 31.1% | 32.5% |
| Mean proportion of state residents with 14 or more “not good” mental health days in the last month | 10.8% | 11.8% |
|  |  |  |
| **Demographic Covariates** |  |  |
| Mean proportion of state residents under 18 | 22.6% | 22.6% |
| Mean proportion of state residents between 18-34 | 20.6% | 20.0% |
| Mean proportion of state residents 65 or older | 15.2% | 16.0% |
| Mean state population density | 229.99 per sq. mile | 155.67 per sq. mile |
| Mean proportion of state residents that identify as White | 70.1% | 76.9% |
| Mean proportion of state residents with at least a Bachelor’s degree | 34.7% | 32.6% |
| Mean proportion of state residents that were born outside of the US | 14.0% | 8.3% |
| Mean proportion of state residents that identify as a veteran | 0.1% | 0.1% |
| **Economic Covariates** |  |  |
| Mean state income | $89,617.11 | $86,435.93 |
| Mean proportion of state residents that are unemployed | 6.4% | 5.4% |
| Proportion of wealth held by a state’s wealthiest decile | 48.8% | 44.7% |
| Mean proportion of state residents living in poverty | 9.0% | 7.9% |
| Mean proportion of state residents that are uninsured | 7.3% | 7.5% |
|  |  |  |
| **Incarceration Prevalence Covariates** |  |  |
| Mean state incarceration rate | 328.30 per 100,000 | 291.16 per 100,000 |
| **Political Covariates** |  |  |
| Mean score of Republican Control of legislative houses and executive branch (minimum 0, maximum 3) | 0.16 | 0.67 |
|  |  |  |
| **COVID-19 Covariates** |  |  |
| Mean state COVID-19 death rate | 72.7 per 100,000 | 71.4 per 100,000 |
| Mean number of weeks spent in COVID-19 lockdowns | 10.00 | 7.84 |
|  |  |  |

Table S7. Predictor Values for Maryland and Synthetic Maryland

| **Covariates** | **Maryland** | **Synthetic Maryland** |
| --- | --- | --- |
| **Firearm Ownership & Policy Covariates** |  |  |
| Proportion of state-months that prohibit firearm possession by those involuntarily committed for inpatient mental health treatment | 100.0% | 60.1% |
| Proportion of state-months that prohibit firearm possession by those involuntarily committed for outpatient mental health treatment | 0.0% | 0.0% |
| Proportion of state-months that prohibit firearm possession by people who have received treatment for alcoholism that exceeds a state-defined threshold | 0.0% | 0.0% |
| Proportion of state-months that prohibit firearm possession by people who have received treatment for alcohol-related problems that exceeds a state-defined threshold | 0.0% | 59.5% |
| Proportion of state-months that prohibit firearm possession by people who have been convicted of a drug-related misdemeanor | 100.0% | 60.1% |
| Proportion of state-months that prohibit firearm possession by people who have been convicted of a felony | 100.0% | 99.4% |
| Proportion of state-months that prohibit firearm possession by people who have committed violent misdemeanors punishable by more than one year of imprisonment | 100.0% | 60.1% |
| Proportion of state-months without a ‘stand your ground’ law | 100.0% | 59.5% |
| Estimated proportion of state residents who own a firearm | 44.1% | 52.3% |
|  |  |  |
| **Behavioral Health Covariates** |  |  |
| Mean proportion of state residents in the upper 80^th^ percentile of self-reported alcohol consumption | 31.3% | 33.1% |
| Mean proportion of state residents with 14 or more “not good” mental health days in the last month | 11.0% | 11.0% |
|  |  |  |
| **Demographic Covariates** |  |  |
| Mean proportion of state residents under 18 | 22.3% | 23.6% |
| Mean proportion of state residents between 18-34 | 20.4% | 19.6% |
| Mean proportion of state residents 65 or older | 15.0% | 14.5% |
| Mean state population density | 622.4 per sq. mile | 96.2 per sq. mile |
| Mean proportion of state residents that identify as White | 54.7% | 73.1% |
| Mean proportion of state residents with at least a Bachelor’s degree | 40.1% | 34.1% |
| Mean proportion of state residents that were born outside of the US | 15.3% | 8.8% |
| Mean proportion of state residents that identify as a veteran | 0.1% | 0.1% |
| **Economic Covariates** |  |  |
| Mean state income | $107,933.30 | $88,634.71 |
| Mean proportion of state residents that are unemployed | 5.5% | 4.9% |
| Proportion of wealth held by a state’s wealthiest decile | 42.6% | 44.9% |
| Mean proportion of state residents living in poverty | 6.4% | 7.7% |
| Mean proportion of state residents that are uninsured | 6.3% | 8.2% |
|  |  |  |
| **Incarceration Prevalence Covariates** |  |  |
| Mean state incarceration rate | 305.6 per 100,000 | 277.6 per 100,000 |
| **Political Covariates** |  |  |
| Mean score of Republican Control of legislative houses and executive branch (minimum 0, maximum 3) | 0.288 | 0.607 |
|  |  |  |
| **COVID-19 Covariates** |  |  |
| Mean state COVID-19 death rate | 64.1 per 100,000 | 75.9 per 100,000 |
| Mean number of weeks spent in COVID-19 lockdowns | 3.12 | 2.70 |
|  |  |  |

Table S8. Predictor Values for Massachusetts and Synthetic Massachusetts

| **Covariates** | **Massachusetts** | **Synthetic Massachusetts** |
| --- | --- | --- |
| **Firearm Ownership & Policy Covariates** |  |  |
| Proportion of state-months that prohibit firearm possession by those involuntarily committed for inpatient mental health treatment | 100.0% | 87.8% |
| Proportion of state-months that prohibit firearm possession by those involuntarily committed for outpatient mental health treatment | 100.0% | 0.0% |
| Proportion of state-months that prohibit firearm possession by people who have received treatment for alcoholism that exceeds a state-defined threshold | 0.0% | 0.0% |
| Proportion of state-months that prohibit firearm possession by people who have received treatment for alcohol-related problems that exceeds a state-defined threshold | 100.0% | 86.3% |
| Proportion of state-months that prohibit firearm possession by people who have been convicted of a drug-related misdemeanor | 100.0% | 86.3% |
| Proportion of state-months that prohibit firearm possession by people who have been convicted of a felony | 100.0% | 100.0% |
| Proportion of state-months that prohibit firearm possession by people who have committed violent misdemeanors punishable by more than one year of imprisonment | 100.0% | 86.3% |
| Proportion of state-months without a ‘stand your ground’ law | 100.0% | 86.3% |
| Estimated proportion of state residents who own a firearm | 21.3% | 46.2% |
|  |  |  |
| **Behavioral Health Covariates** |  |  |
| Mean proportion of state residents in the upper 80^th^ percentile of self-reported alcohol consumption | 35.6% | 33.2% |
| Mean proportion of state residents with 14 or more “not good” mental health days in the last month | 12.0% | 10.6% |
|  |  |  |
| **Demographic Covariates** |  |  |
| Mean proportion of state residents under 18 | 20.0% | 23.2% |
| Mean proportion of state residents between 18-34 | 23.2% | 18.4% |
| Mean proportion of state residents 65 or older | 16.2% | 15.6% |
| Mean state population density | 879.2 per sq. mile | 82.5 per sq. mile |
| Mean proportion of state residents that identify as White | 76.9% | 81.8% |
| Mean proportion of state residents with at least a Bachelor’s degree | 43.7% | 35.3% |
| Mean proportion of state residents that were born outside of the US | 16.8% | 8.2% |
| Mean proportion of state residents that identify as a veteran | 0.1% | 0.1% |
| **Economic Covariates** |  |  |
| Mean state income | $108,784.80 | $89,687.56 |
| Mean proportion of state residents that are unemployed | 5.3% | 4.2% |
| Proportion of wealth held by a state’s wealthiest decile | 54.6% | 45.2% |
| Mean proportion of state residents living in poverty | 7.1% | 6.4% |
| Mean proportion of state residents that are uninsured | 2.8% | 4.9% |
|  |  |  |
| **Incarceration Prevalence Covariates** |  |  |
| Mean state incarceration rate | 142.6 per 100,000 | 202.8 per 100,000 |
| **Political Covariates** |  |  |
| Mean score of Republican Control of legislative houses and executive branch (minimum 0, maximum 3) | 0.29 | 0.43 |
|  |  |  |
| **COVID-19 Covariates** |  |  |
| Mean state COVID-19 death rate | 62.9 per 100,000 | 73.16 per 100,000 |
| Mean number of weeks spent in COVID-19 lockdowns | 3.1 | 3.2 |
|  |  |  |

Table S9. Predictor Values for Oregon and Synthetic Oregon

| **Covariates** | **Oregon** | **Synthetic Oregon** |
| --- | --- | --- |
| **Firearm Ownership & Policy Covariates** |  |  |
| Proportion of state-months that prohibit firearm possession by those involuntarily committed for inpatient mental health treatment | 100.0% | 91.4% |
| Proportion of state-months that prohibit firearm possession by those involuntarily committed for outpatient mental health treatment | 87.5% | 47.4% |
| Proportion of state-months that prohibit firearm possession by people who have received treatment for alcoholism that exceeds a state-defined threshold | 0.0% | 6.9% |
| Proportion of state-months that prohibit firearm possession by people who have received treatment for alcohol-related problems that exceeds a state-defined threshold | 0.0% | 41.4% |
| Proportion of state-months that prohibit firearm possession by people who have been convicted of a drug-related misdemeanor | 0.0% | 44.0% |
| Proportion of state-months that prohibit firearm possession by people who have been convicted of a felony | 100.0% | 83.1% |
| Proportion of state-months that prohibit firearm possession by people who have committed violent misdemeanors punishable by more than one year of imprisonment | 100.0% | 50.9% |
| Proportion of state-months without a ‘stand your ground’ law | 100.0% | 41.4% |
| Estimated proportion of state residents who own a firearm | 0.528 | 52.7% |
|  |  |  |
| **Behavioral Health Covariates** |  |  |
| Mean proportion of state residents in the upper 80^th^ percentile of self-reported alcohol consumption | 34.9% | 32.6% |
| Mean proportion of state residents with 14 or more “not good” mental health days in the last month | 13.7% | 12.0% |
|  |  |  |
| **Demographic Covariates** |  |  |
| Mean proportion of state residents under 18 | 20.9% | 22.6% |
| Mean proportion of state residents between 18-34 | 20.2% | 19.5% |
| Mean proportion of state residents 65 or older | 17.3% | 16.4% |
| Mean state population density | 43.06 per sq. mile | 104.30 per sq. mile |
| Mean proportion of state residents that identify as White | 82.9% | 79.4% |
| Mean proportion of state residents with at least a Bachelor’s degree | 33.6% | 31.2% |
| Mean proportion of state residents that were born outside of the US | 9.9% | 8.4% |
| Mean proportion of state residents that identify as a veteran | 0.1% | 0.1% |
| **Economic Covariates** |  |  |
| Mean state income | $82,180.30 | $83,185.97 |
| Mean proportion of state residents that are unemployed | 5.9% | 5.3% |
| Proportion of wealth held by a state’s wealthiest decile | 47.3% | 45.1% |
| Mean proportion of state residents living in poverty | 8.5% | 8.4% |
| Mean proportion of state residents that are uninsured | 7.1% | 7.6% |
|  |  |  |
| **Incarceration Prevalence Covariates** |  |  |
| Mean state incarceration rate | 351.4 per 100,000 | 353.4 per 100,000 |
| **Political Covariates** |  |  |
| Mean score of Republican Control of legislative houses and executive branch (minimum 0, maximum 3) | 0 | 0.73 |
|  |  |  |
| **COVID-19 Covariates** |  |  |
| Mean state COVID-19 death rate | 43.1 per 100,000 | 77.1 per 100,000 |
| Mean number of weeks spent in COVID-19 lockdowns | 4.16 | 3.03 |
|  |  |  |

Table S10. Predictor Values for Rhode Island and Synthetic Rhode Island

| **Covariates** | **Rhode Island** | **Synthetic Rhode Island** |
| --- | --- | --- |
| **Firearm Ownership & Policy Covariates** |  |  |
| Proportion of state-months that prohibit firearm possession by those involuntarily committed for inpatient mental health treatment | 100.0% | 97.0% |
| Proportion of state-months that prohibit firearm possession by those involuntarily committed for outpatient mental health treatment | 100.0% | 20.7% |
| Proportion of state-months that prohibit firearm possession by people who have received treatment for alcoholism that exceeds a state-defined threshold | 0.0% | 0.0% |
| Proportion of state-months that prohibit firearm possession by people who have received treatment for alcohol-related problems that exceeds a state-defined threshold | 0.0% | 26.7% |
| Proportion of state-months that prohibit firearm possession by people who have been convicted of a drug-related misdemeanor | 0.0% | 54.6% |
| Proportion of state-months that prohibit firearm possession by people who have been convicted of a felony | 0.0% | 51.5% |
| Proportion of state-months that prohibit firearm possession by people who have committed violent misdemeanors punishable by more than one year of imprisonment | 0.0% | 54.6% |
| Proportion of state-months without a ‘stand your ground’ law | 100.0% | 26.7% |
| Estimated proportion of state residents who own a firearm | 28.1% | 50.7% |
|  |  |  |
| **Behavioral Health Covariates** |  |  |
| Mean proportion of state residents in the upper 80^th^ percentile of self-reported alcohol consumption | 33.9% | 31.5% |
| Mean proportion of state residents with 14 or more “not good” mental health days in the last month | 12.9% | 12.4% |
|  |  |  |
| **Demographic Covariates** |  |  |
| Mean proportion of state residents under 18 | 19.6% | 23.3% |
| Mean proportion of state residents between 18-34 | 22.1% | 20.4% |
| Mean proportion of state residents 65 or older | 16.9% | 15.8% |
| Mean state population density | 1,027.4 per sq. mile | 173.9 per sq. mile |
| Mean proportion of state residents that identify as White | 79.13% | 81.78% |
| Mean proportion of state residents with at least a Bachelor’s degree | 33.93% | 32.00% |
| Mean proportion of state residents that were born outside of the US | 13.77% | 6.96% |
| Mean proportion of state residents that identify as a veteran | 0.06% | 0.06% |
| **Economic Covariates** |  |  |
| Mean state income | $86,582.53 | $84,210.44 |
| Mean proportion of state residents that are unemployed | 6.07% | 4.83% |
| Proportion of wealth held by a state’s wealthiest decile | 46.11% | 44.27% |
| Mean proportion of state residents living in poverty | 8.45% | 7.70% |
| Mean proportion of state residents that are uninsured | 4.85% | 6.93% |
|  |  |  |
| **Incarceration Prevalence Covariates** |  |  |
| Mean state incarceration rate | 162.9 per 100,000 | 311.4 per 100,000 |
| **Political Covariates** |  |  |
| Mean score of Republican Control of legislative houses and executive branch (minimum 0, maximum 3) | 0 | 0.79 |
|  |  |  |
| **COVID-19 Covariates** |  |  |
| Mean state COVID-19 death rate | 68.6 per 100,000 | 67.6 per 100,000 |
| Mean number of weeks spent in COVID-19 lockdowns | 3.1 | 2.4 |
|  |  |  |

Table S11. Predictor Values for Vermont and Synthetic Vermont

| **Covariates** | **Vermont** | **Synthetic Vermont** |
| --- | --- | --- |
| **Firearm Ownership & Policy Covariates** |  |  |
| Proportion of state-months that prohibit firearm possession by those involuntarily committed for inpatient mental health treatment | 0.0% | 40.6% |
| Proportion of state-months that prohibit firearm possession by those involuntarily committed for outpatient mental health treatment | 0.0% | 0.0% |
| Proportion of state-months that prohibit firearm possession by people who have received treatment for alcoholism that exceeds a state-defined threshold | 0.0% | 0.0% |
| Proportion of state-months that prohibit firearm possession by people who have received treatment for alcohol-related problems that exceeds a state-defined threshold | 0.0% | 40.6% |
| Proportion of state-months that prohibit firearm possession by people who have been convicted of a drug-related misdemeanor | 0.0% | 40.6% |
| Proportion of state-months that prohibit firearm possession by people who have been convicted of a felony | 0.0% | 50.5% |
| Proportion of state-months that prohibit firearm possession by people who have committed violent misdemeanors punishable by more than one year of imprisonment | 0.0% | 40.6% |
| Proportion of state-months without a ‘stand your ground’ law | 100.0% | 44.3% |
| Estimated proportion of state residents who own a firearm | 0.549 | 54.3% |
|  |  |  |
| **Behavioral Health Covariates** |  |  |
| Mean proportion of state residents in the upper 80^th^ percentile of self-reported alcohol consumption | 37.1% | 33.7% |
| Mean proportion of state residents with 14 or more “not good” mental health days in the last month | 12.5% | 11.0% |
|  |  |  |
| **Demographic Covariates** |  |  |
| Mean proportion of state residents under 18 | 18.7% | 22.5% |
| Mean proportion of state residents between 18-34 | 19.0% | 18.2% |
| Mean proportion of state residents 65 or older | 18.9% | 18.2% |
| Mean state population density | 68.1 per sq. mile | 37.6 per sq. mile |
| Mean proportion of state residents that identify as White | 93.8% | 85.9% |
| Mean proportion of state residents with at least a Bachelor’s degree | 38.5% | 33.4% |
| Mean proportion of state residents that were born outside of the US | 4.15% | 7.1% |
| Mean proportion of state residents that identify as a veteran | 0.1% | 0.1% |
| **Economic Covariates** |  |  |
| Mean state income | $79,709.99 | $80,824.52 |
| Mean proportion of state residents that are unemployed | 4.0% | 4.0% |
| Proportion of wealth held by a state’s wealthiest decile | 42.3% | 43.6% |
| Mean proportion of state residents living in poverty | 6.7% | 7.1% |
| Mean proportion of state residents that are uninsured | 4.2% | 7.1% |
|  |  |  |
| **Incarceration Prevalence Covariates** |  |  |
| Mean state incarceration rate | 183.6 per 100,000 | 283.2 per 100,000 |
| **Political Covariates** |  |  |
| Mean score of Republican Control of legislative houses and executive branch (minimum 0, maximum 3) | 20.6% | 57.7% |
|  |  |  |
| **COVID-19 Covariates** |  |  |
| Mean state COVID-19 death rate | 23.5 per 100,000 | 72.5 per 100,000 |
| Mean number of weeks spent in COVID-19 lockdowns | 3.1 | 2.3 |
|  |  |  |

Table S12. Predictor Values for Washington and Synthetic Washington

| **Covariates** | **Washington** | **Synthetic Washington** |
| --- | --- | --- |
| **Firearm Ownership & Policy Covariates** |  |  |
| Proportion of state-months that prohibit firearm possession by those involuntarily committed for inpatient mental health treatment | 100.0% | 82.7% |
| Proportion of state-months that prohibit firearm possession by those involuntarily committed for outpatient mental health treatment | 100.0% | 31.0% |
| Proportion of state-months that prohibit firearm possession by people who have received treatment for alcoholism that exceeds a state-defined threshold | 0.0% | 0.0% |
| Proportion of state-months that prohibit firearm possession by people who have received treatment for alcohol-related problems that exceeds a state-defined threshold | 0.0% | 46.8% |
| Proportion of state-months that prohibit firearm possession by people who have been convicted of a drug-related misdemeanor | 0.0% | 46.8% |
| Proportion of state-months that prohibit firearm possession by people who have been convicted of a felony | 100.0% | 100.0% |
| Proportion of state-months that prohibit firearm possession by people who have committed violent misdemeanors punishable by more than one year of imprisonment | 0.0% | 46.8% |
| Proportion of state-months without a ‘stand your ground’ law | 100.0% | 46.8% |
| Estimated proportion of state residents who own a firearm | 48.7% | 52.0% |
|  |  |  |
| **Behavioral Health Covariates** |  |  |
| Mean proportion of state residents in the upper 80^th^ percentile of self-reported alcohol consumption | 32.8% | 32.6% |
| Mean proportion of state residents with 14 or more “not good” mental health days in the last month | 12.3 | 11.2% |
|  |  |  |
| **Demographic Covariates** |  |  |
| Mean proportion of state residents under 18 | 22.2% | 23.7% |
| Mean proportion of state residents between 18-34 | 20.7% | 19.9% |
| Mean proportion of state residents 65 or older | 15.2% | 15.2% |
| Mean state population density | 111.6 per sq. mile | 58.9 per sq. mile |
| Mean proportion of state residents that identify as White | 74.3% | 77.3% |
| Mean proportion of state residents with at least a Bachelor’s degree | 35.9% | 32.9% |
| Mean proportion of state residents that were born outside of the US | 14.3% | 9.8% |
| Mean proportion of state residents that identify as a veteran | 0.1% | 0.1% |
| **Economic Covariates** |  |  |
| Mean state income | $97,129.62 | $87,707.75 |
| Mean proportion of state residents that are unemployed | 5.3% | 5.2% |
| Proportion of wealth held by a state’s wealthiest decile | 48.0% | 44.8% |
| Mean proportion of state residents living in poverty | 7.1% | 7.8% |
| Mean proportion of state residents that are uninsured | 6.7% | 8.2% |
|  |  |  |
| **Incarceration Prevalence Covariates** |  |  |
| Mean state incarceration rate | 238.9 per 100,000 | 312.8 per 100,000 |
| **Political Covariates** |  |  |
| Mean score of Republican Control of legislative houses and executive branch (minimum 0, maximum 3) | 0.08 | 0.67 |
|  |  |  |
| **COVID-19 Covariates** |  |  |
| Mean state COVID-19 death rate | 43.3 per 100,000 | 76.1 per 100,000 |
| Mean number of weeks spent in COVID-19 lockdowns | 4.1 | 2.8 |
|  |  |  |

Table S13: ERPO Petition Volume by State Year

| **State** | **Year** | **ERPO Policy Active** | **Total ERPOs Petitioned** |
| --- | --- | --- | --- |
| California | 2018 | Yes | 424 |
|  | 2019 | Yes | 1110 |
|  | 2020 | Yes | 1284 |
|  | 2021 | Yes | 1384 |
|  | 2022 | Yes | 1909 |
| Delaware | 2018 | No | 0 |
|  | 2019 | Yes | 24 |
|  | 2020 | Yes | 16 |
|  | 2021 | Yes | 29 |
|  | 2022 | Yes | 13 |
| Florida | 2018 | Yes | 1192 |
|  | 2019 | Yes | 2075 |
|  | 2020 | Yes | 2309 |
|  | 2021 | Yes | 2482 |
|  | 2022 | Yes | 2907 |
| Illinois | 2018 | No | 0 |
|  | 2019 | Yes | 74 |
|  | 2020 | Yes | 67 |
|  | 2021 | Yes | 38 |
|  | 2022 | Yes | 112 |
| Maryland | 2018 | Yes | 303 |
|  | 2019 | Yes | 873 |
|  | 2020 | Yes | 712 |
|  | 2021 | Yes | 754 |
|  | 2022 | Yes | 741 |
| Massachusetts | 2018 | Yes | 10 |
|  | 2019 | Yes | 19 |
|  | 2020 | Yes | 9 |
|  | 2021 | Yes | 7 |
|  | 2022 | Yes | 12 |
| Oregon | 2018 | Yes | 74 |
|  | 2019 | Yes | 116 |
|  | 2020 | Yes | 144 |
|  | 2021 | Yes | 149 |
|  | 2022 | Yes | 166 |
| Rhode Island | 2018 | Yes | 17 |
|  | 2019 | Yes | 48 |
|  | 2020 | Yes | 48 |
|  | 2021 | Yes | 46 |
|  | 2022 | Yes | 75 |
| Vermont | 2018 | Yes | 38 |
|  | 2019 | Yes | 38 |
|  | 2020 | Yes | 31 |
|  | 2021 | Yes | 30 |
|  | 2022 | Yes | 22 |
| Virginia | 2018 | No | 0 |
|  | 2019 | No | 0 |
|  | 2020 | Yes | 44 |
|  | 2021 | Yes | 131 |
|  | 2022 | Yes | 189 |
| Washington | 2018 | Yes | 147 |
|  | 2019 | Yes | 161 |
|  | 2020 | Yes | 213 |
|  | 2021 | Yes | 187 |
|  | 2022 | Yes | 132 |

Accompanying Figure:


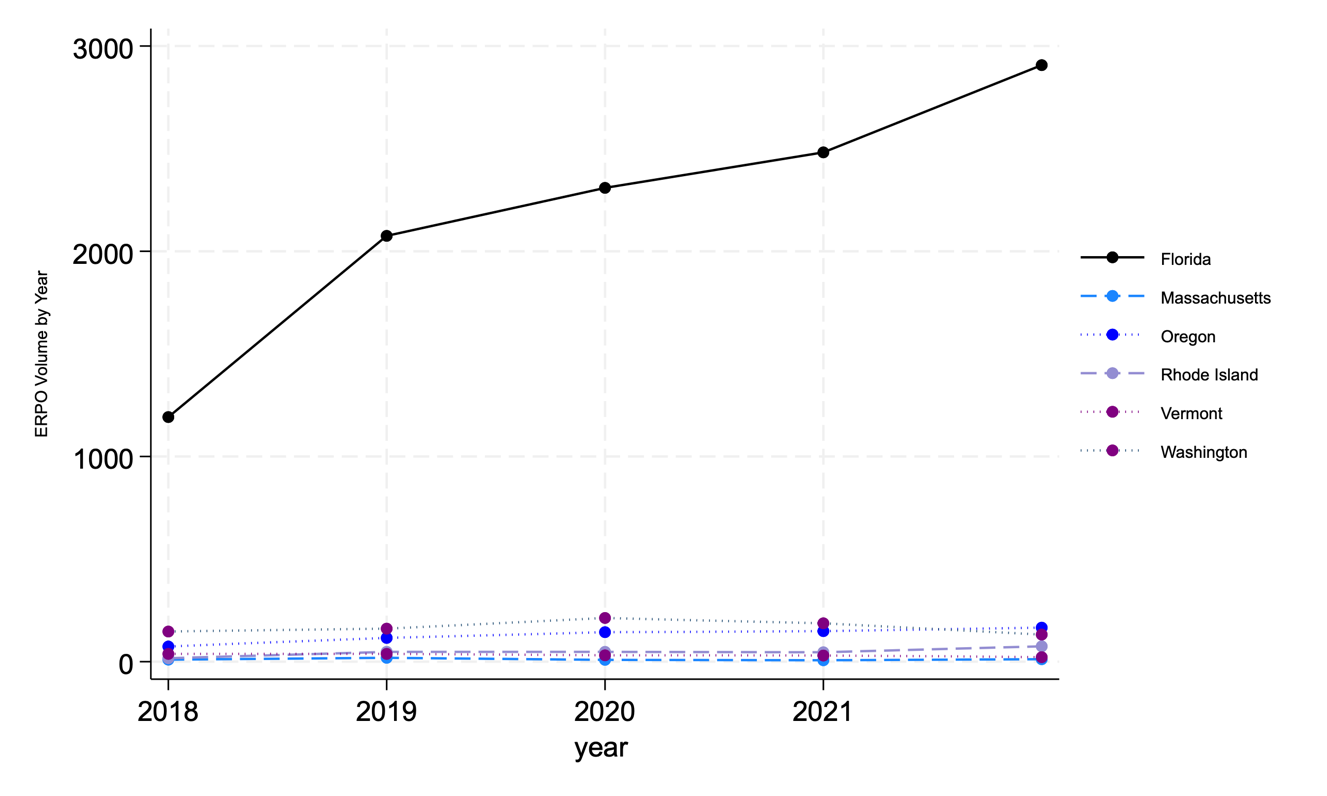

Supplement: Supplementary file 1 — Supplementary Material 1. [file 40621_2026_663_MOESM1_ESM.docx]
